# Greener Method for the Application of TiO_2_ Nanoparticles to Remove Herbicide in Water

**DOI:** 10.1155/2023/3806240

**Published:** 2023-07-11

**Authors:** Hoang Hiep, Pham Tuan Anh, Van-Duong Dao, Dang Viet Quang

**Affiliations:** ^1^Academy for Green Growth, Vietnam National University of Agriculture, Gia Lam, Hanoi, Vietnam; ^2^Falcuty of Biotechnology, Chemistry and Environmental Engineering, Phenikaa University, Hanoi 12116, Vietnam

## Abstract

TiO_2_ nanoparticles have emerged as a great photocatalyst to degrade organic contaminants in water; however, the nanoparticles dispersed in water could be difficult to be recovered and potentially become contaminant. Herbicide like 2,4-dichlorophenoxyacetic acid (2,4-D) used in agriculture usually ends up with a large fraction remaining in water and sediment, which may cause potential risk to human health and the ecosystem. This study proposes a greener method to utilize TiO_2_ as photocatalyst to remove 2,4-D from water. Accordingly, TiO_2_ nanoparticles (10–45 nm) were synthesized and grafted on lightweight fired clay to generate a TiO_2_-based floating photocatalyst. Experimental testing revealed that 60.2% of 2,4-D (0.1 mM) can be decomposed in 250 min under UV light with TiO_2_-grafted lightweight fired clay floating on water. Degradation fits well into the pseudo-first-order kinetic model. The floating photocatalysts can degrade approximately 50% 2,4-D in 250 min under sunlight and the degradation efficiency is stable for cycles. The results revealed that the fabrication of floating photocatalyst could be a promising and greener way to remove herbicide contaminants in water using TiO_2_.

## 1. Introduction

2,4-dichlorophenoxyacetic acid (2,4-D) is an herbicide agent that has been widely used to control broadleaf weeds in agriculture and urban landscape practices [1]. This chemical has been registered as an active ingredient in approximately 1500 herbicide formulations, with a large amount being produced and consumed worldwide every year [2]. In China, for example, 2,4-D production reached 40,000 tons/year in 2010 [3]; meanwhile, the consumption in the USA was about 13,000–15,000 tons annually in 2001 [2]. Herbicides are usually applied onto soil or sprayed over crops; therefore, they can reach superficial water and sediments [4]. It is estimated that 91.7% of 2,4-D ends up in surface and ground water due to its high solubility in water [5]. 2,4-D is a moderately persistent chemical, which can be decomposed by both photodegradation and microbial degradation at a very slow rate, with a half-life between 20 and 312 days [6]. 2,4-D contamination could be the source of health hazard to exposed animals and human, which may cause the endocrine disruption, reproductive disorder, genetic alterations, and carcinogenic effects [1]. Because of these environmental and health concerns, it is necessary to eliminate 2,4-D from water.

Photolysis and microbial degradation can occur naturally, however, at a slow rate, thus, it requires effective treatment technologies for the elimination of 2,4-D [4]. Removal of 2,4-D from water has been investigated with various treatment technologies such as adsorption [7–12], photo/oxidation [13, 14], electrochemical oxidation [15, 16], photocatalysis [17–22], and microbial degradation [23, 24]. In general, these technologies showed relatively high efficiency in the removal of 2,4-D from contaminated water. Activated carbon and metal organic frameworks, for example, can adsorb up to 352.9 mg/g and 556 mg/g, respectively [9, 11]. A photocatalyst based on TiO_2_ can degrade 83% 2,4-D in 180 min under visible light. Electrochemical oxidation posed as a great treatment process that can reach 95.9% removal efficiency [15]. Even though these technologies efficiently remove 2,4-D, they may not be suitable for treatment of scattered or large water resources such as lake, river, irrigated water, and aquaculture water. These required more sustainable and cost-effective treatment technologies.

Floating photocatalyst-based water treatment technology (FPWT) that uses sunlight to breakdown pollutants has recently attracted great attention because of its potential for large-scale application, particularly to treat water resources that are contaminated with persistent organic pollutants (POPs) such as herbicides, pesticides, and antibiotics [25, 26]. These pollutants, which usually originated from agriculture, aquaculture, livestock, medicine, and chemical industries enter water reservoir and could not be removed by normal water treatment plants [1, 27, 28]. FPWT breaks POPs under sunlight using photocatalysts that are grafted on a floating substrate. When the floating photocatalysts (FPC) are dispatched to a water reservoir, it will float and continue degrading POPs under sunlight irradiation without any requirement of external intervention.

Several substrates including synthetic polymers [29–32], polymer composites [33–37], wood [38], ceramics and silica [39, 40], natural light-weight stones [41–43], and concrete [44] have been used to support photocatalysts. Testing in a laboratory revealed that FPWT effectively removes a variety of organic pollutants. TiO_2_-PANI/cork FPC can decompose 95.2% methyl orange, 85.3% 4-nitrophenol, and over 60% of phenol, 2,4-dinitrophenol, toluidine, salicylic acid, and benzoic acid in 210 min under sunlight [33]. Ni-N-TiO_2_ expanded graphite composite FPC removed 96.9% diesel oil after 5 h irradiation by visible light [34]. 98.1% methyl blue can be removed by B-N-TiO_2_/expanded perlite FPC after 5 h under visible light [45]. 94.8% Congo red can be removed by TiO_2_-loaded palm trunk after 150 h of irradiation by sunlight [38]. FPC also showed effective removal of NH_3_ and naphthenic acids [25, 39].

Previous studies revealed that TiO_2_ can effectively decompose 2,4-D in water [46, 47]. The decomposition efficiency may vary with the TiO_2_ composition; pure anatase TiO_2_ can remove 68.2–70.5%, but it increased to 92.7% as TiO_2_ containing 8% rutile [46]. Obviously, TiO_2_ powder can effectively remove 2,4-D; however, it could not be directly dispersed into water resources since it will require very large amount and could not be recovered. TiO_2_ powder, consequently, is not feasible for the direct use to treat organic contaminants in large water reservoirs. Thus, the fabrication of FPC could be a greener and more feasible measure for the removal of 2,4-D and other organic pollutants from water using TiO_2_. To the best of our knowledge, the application of FPC for the degradation of 2,4-D has not been investigated; therefore, the objective of this work is to graft TiO_2_ onto LFC for TiO_2_-based FPC production and investigate its potential for 2,4-D removal.

## 2. Experimental

### 2.1. Materials

Clay samples from Phu Tho province with the composition shown in Table 1 were purchased from a local supplier. Rice husk was collected from a local source in Hai Duong, Viet Nam. Clay samples were dried and ground to the particle size ≤63 *μ*m while rice husk was crushed until the size of ≤0.5 mm. Tetraisopropyl orthotitanate (TTIP, 97%), isopropanol (IPA, 99.5%), acetyl acetone (ACAC, 99%), and 2,4-dichlorophenoxyacetic acid (2,4-D) were purchased from Sigma-Aldrich and used without further purification.

### 2.2. Preparation of Lightweight Fired Clay

LFC was prepared in accordance with previous publications [48, 49]. In a typical preparation process, desired amounts of clay and rice husk with a mass ratio of 1 : 1 were weighed and mixed well prior to the addition of water. The water quantity was sufficiently adjusted to ensure the plasticity of the clay mixture. The clay mixture was pelletized into spherical-like granules, which were then dried under sunlight for 2-3 days before being fired in a furnace. The firing process was conducted in two steps from the room temperature to 1200°C. The first step related to the temperature increment from the room temperature to 200°C at the ramping rate of 15°C/min and then to 1200°C at the ramping rate of 20°C/min. The temperature remained constant for 20 min and 10 min at the end of the first and second steps, respectively. After cooling down to room temperature, the LFC sample was stored for further characterization and experiments.

### 2.3. TiO_2_ Synthesis

TiO_2_ was prepared by a hydrothermal method adapted from [50] using tetra-isopropyl orthotitanate as a titanium precursor. Typically, a mixture of TTIP : ACA : IPA with a molar ratio of 1 : 1 : 30 was prepared by slow addition of TTIP into a 500 mL beaker containing ACA and IPA, followed by the introduction of a solution of 15 wt% water in IPA. The mixture was continuously stirred at room temperature for 30 min, transferred to a 500 mL hydrothermal reactor made of Teflon-lined stainless steel. The mixture was then hydrothermally treated by placing the reactor in an oven at 160°C for 9 h. Solid TiO_2_ was separated and washed with plenty of ethanol and water by centrifugation. The obtained TiO_2_ was dried at 90°C for 24 h for later characterization and fabrication of FPC.

### 2.4. Preparation of Floating Photocatalyst

FPC that is TiO_2_-modified lightweight fired clay (TiO_2_/LFC) was prepared according to a procedure described elsewhere [39]. First, 5 g TiO_2_ was dispersed into 150 mL ethanol in a 500 mL beaker, followed by the adjustment of pH to ∼3.5 with dilute HNO_3_. The mixture was sonicated for 30 min to generate a homogenous slurry, which was then gently mixed with 20 g of LFC granules in 2 h for TiO_2_ to adsorb onto LFC. Subsequently, LFC granules were separated and dried in the oven for 2 h at 120°C prior to calcination at 450°C in 30 min. Finally, to remove any TiO_2_ particles that were not grafted on the LFC surface, LFC granules were washed with distilled water for several times and again dried at 120°C for 2 h.

### 2.5. Photocatalytic Degradation Tests

Photocatalytic degradation was tested under UV light by a batch-wise method in an experimental chamber consists of a 6-place magnetic stirrer at the bottom and 10 fluorescent UV lamps (G8 W T5 from Sylvania producer with *λ*_max_ = 365 nm to 8 watt) mounted on the top. Over the magnetic stirrer, the energy density of 6.5 mW/cm^2^ was determined by using a UVA-B light meter and an ILT 1400-A Radiometer Photometer. The chamber was constructed mainly by aluminium material and was completely covered by aluminium foil during testing. A similar experimental setup was used for the sunlight test; however, the chamber with UV light was removed for sunlight irradiation.

In a typical experiment, 0.5 g FPC and 50 mL of 2,4-D 0.1 mM solution (catalyst dose: 10 g/L) were added into a 250 mL beaker, stirred on a magnetic stirrer in the experimental chamber, and then the UV light was turned on. To follow the degradation progress, samples were extracted after a certain duration, filtered, and analyzed for 2,4-D concentration. Two FPC granule samples with average sizes of 5 mm and 8 mm were tested to evaluate the potential effect of granular size on its catalytic degradation activity. Blank and control experiments were conducted in the same procedure with no catalyst, pure TiO_2_ (0.6 g/L) or LFC substrate (10 g/L). To study the recyclability of photocatalyst, FPC (8 mm) was recovered after the experiment, slightly washed with water, dried at 120°C for 2 h, and then reused in another cycle to examine any possible decrease in photocatalytic activities.

To conduct radical scavenging experiments, three radical scavengers, i.e., benzoquinone, EDTA, and isopropanol were captured to capture O^−^_2_, h^+^, and OH, respectively. Accordingly, each scavenger was added to a beaker containing 2,4-D solution with FPC (8 mm), which was then placed in a UV chamber for 300 min and samples were taken for analyses. To further confirm the 2,4-D degradation, experiments were conducted 2,4-D solution (5 ppm) without scavenger and samples were collected for the analyses of total organic carbon.

### 2.6. Characterization

The specific surface areas of samples were analyzed by nitrogen adsorption/desorption method using Micromeritics TriStar II Plus. Samples were degassed at 250°C for 5 h prior to analysis and the surface area was determined by the BET method. X-ray diffraction (XRD) patterns were collected on the XRD D8 Advance Bruker using a Cu-K*α* source. Scanning electron microscopy (SEM) images were observed on the JEOL 7500F coupled with energy-dispersive X-ray spectroscopy. Fourier transformer infrared spectroscopic studies were conducted on the FTIR 6300 spectrometer (Jasco). 2,4-D concentration was analyzed on an HPLC 5890 series II, Shimadzu using a UV detector at 285 nm, a Zipax SAX (duPont) C18 column, and solvent system including CH_3_CN (A, 60%) and H_2_O with 0.15% acetic acid (B, 40%) at a flow rate of 1 ml/min and an injection volume of 20 *μ*l. Total organic carbon was analyzed on TOC Veolia/Suez Sievers M5310C Laboratory.

## 3. Results and Discussion

### 3.1. Material Characterization

**Figure 1:**
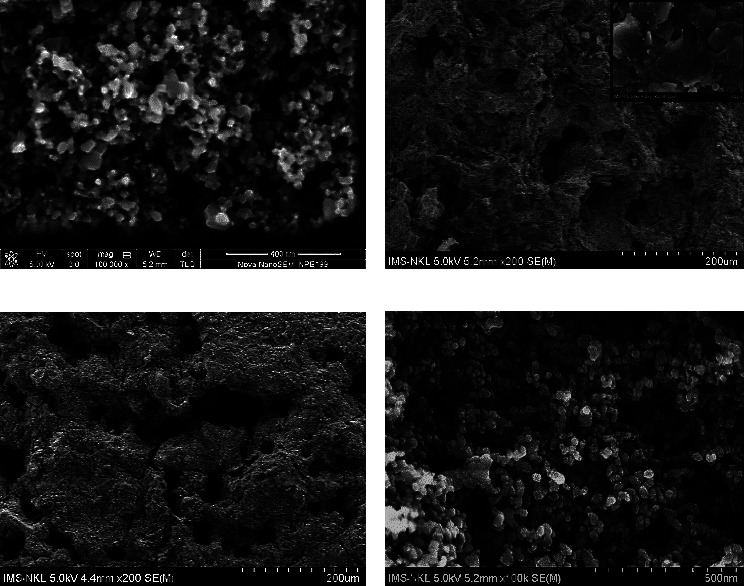
Representative SEM images of TiO_2_ (a), LFC surface ((b) and higher magnification (50k) b, inset), TiO_2_-coated LFC (low magnification, (c)), and TiO_2_-coated LFC (high magnification, (d)).

**Figure 2:**
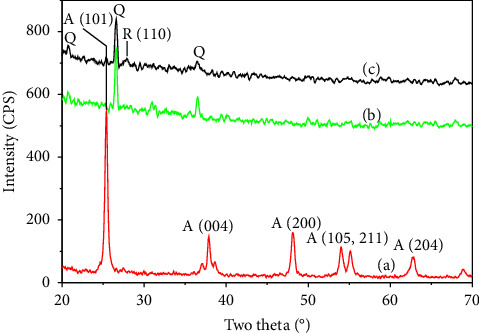
XRD patterns of TiO_2_ (a), LFC (b), and TiO_2_-coated LFC (c).

TiO_2_ photocatalyst and LFC floating substrate were prepared separately, and then TiO_2_ was grafted on the LFC surface by an adsorption-calcination procedure without the addition of any binder. TiO_2_ was prepared by a hydrothermal technique using TTIP as a titanium precursor. This method allows one to synthesize anatase or anatase/rutile mixed TiO_2_ particles at relatively mild condition [50, 51]. The coexistence of the anatase/rutile phase reduces the band gap that enhances the photocatalytic activity of TiO_2_ in the range of visible light [52, 53]. After hydrothermal treatment, TiO_2_ nanoparticles were obtained with the particle size ranging from 10 to 45 nm. These particles tended to agglomerate into mesoporous powder, as shown in Figure 1(a). To evaluate the crystalline phases of TiO_2_, an XRD pattern was collected and analyzed (Figure 2(a)). The resulting TiO_2_ has a tetragonal structure of anatase corresponding to a PDF number of 01-078-2486. Characteristic diffraction at 25.3, 37.9, 48.1, and 62.9° are, respectively, assigned to the (101), (004), (200), and (204) crystal planes of anatase TiO_2_.

Representative SEM images of the LFC surface are shown in Figure 1(b). LFC has a porous structure in which large pores can reach a size of ≈100 *μ*m. Its highly porous structure gives it a low bulk density (<1 g/cm^3^). Higher magnification (Figure 1(b) inset) revealed that LFC constitutes of laminar structure of silicate that were interconnected into a highly porous network, similar observation in previous studies [54, 55]. This type of materials shows relatively good adsorption performance [54, 56]. Thus, the LFC surface was almost completely covered by TiO_2_ nanoparticles as soon as it was contacted with TiO_2_ slurry (Figure 1(c)). After calcination at, more and larger cracks appeared on the surface of the layer; however, the microstructure of TiO_2_ was unchanged (Figure 1(d)). The interconnected TiO_2_ nanoparticles percolated into pores and were deposited onto the LFC surface to form a porous layer. The degree of TiO_2_ nanoparticle aggregation in the porous layer looks similar to that in the original TiO_2_ powder. This restricted the accessibility to pores in the LFC structure, which resulted in a significant reduction in the surface area of LFC from 37.7 to 1.2 m^2^/g.

The presence of TiO_2_ on LFC was asserted by the XRD study, as shown in (Figure 2). The XRD pattern of LFC exhibited peaks at 20.6, 26.5, 36.5, and 40.2°, which could be attributed to the diffraction of quartz. Diffraction at 30.9 and 40.8° corresponds to the mullite phase, which was upon the calcination. Most of these peaks decreased when LFC adsorbed TiO_2_ slurry and calcined, except for the peak at 26.5° that belongs to the stable quartz phase. In addition, a novel and distinct peak emerged at 25.3° and some minor peaks at 37.9, and 48.1° that could be assigned to the characteristic diffraction of anatase TiO_2_. This indicated that the TiO_2_ was successfully grafted onto the LFC. Moreover, several additional minor peaks were observed at 27.7° on the calcined samples, which suggest the possible transformation of anatase to rutile TiO_2_ during calcination.

**Figure 3:**
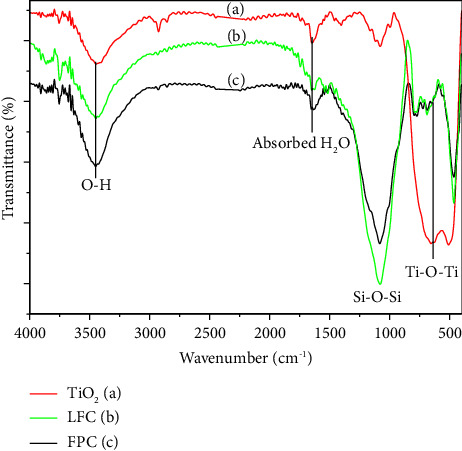
FTIR spectra of as-synthesized TiO_2_ (a), LFC (b), and resulting floating photocatalyst (c).

**Figure 4:**
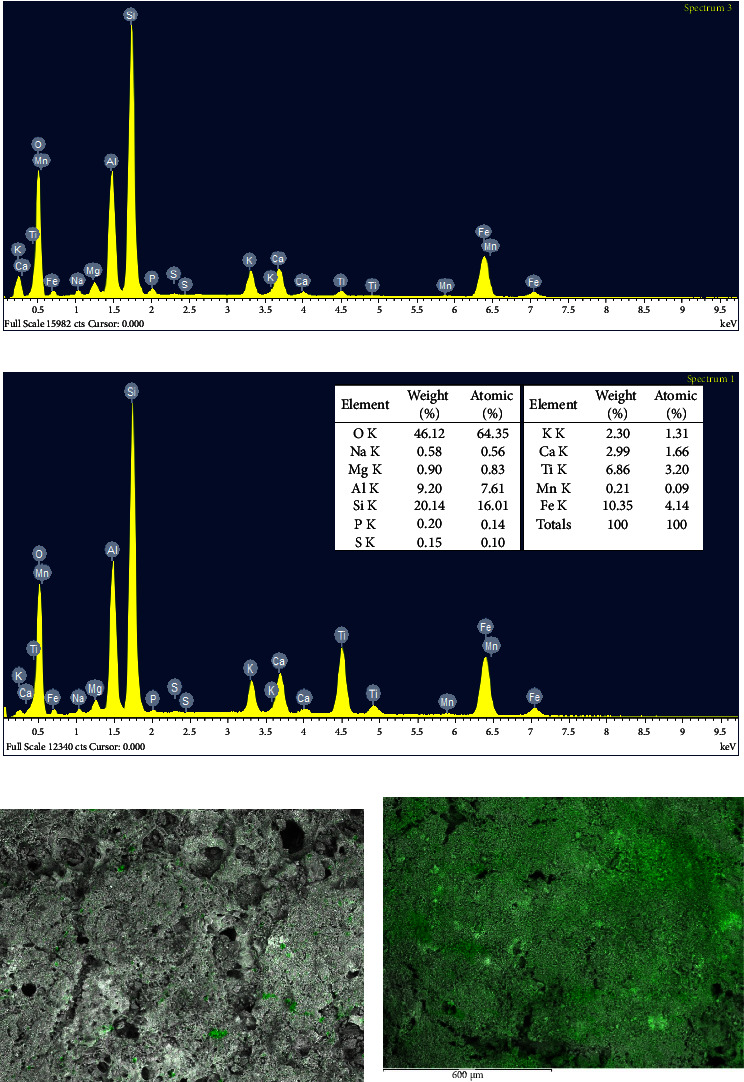
EDX spectra and elemental composition of LFC (a), TiO_2_-coated LFC (b), the elemental mapping of titanium on LFC (c), and TiO_2_-coated LFC (d).

The addition of TiO_2_ onto LFC was further observed on the FTIR spectra of the samples. As shown in Figure 3, the vibration band at ∼3400 cm^−1^ and 1630 cm^−1^ attributed to O-H groups in the structure of TiO_2_, LFC, and FPC and adsorbed water, respectively. The characteristic Si-O-Si bond (∼1080 cm^−1^) of the LFC structure was remained after attachment of TiO_2_. Particularly, the distinct vibration band belonging to the Ti-O-Ti bond (654.7 cm^−1^) of anatase TiO_2_ was reduced significantly but was detectable on the spectrum of FPC. This further confirmed the formation of the TiO_2_ layer on the surface of the LFC. Elemental analyses by EDX indicated that the content of Ti increased from 0.82 wt% to 6.86 wt% after TiO_2_ was grafted on the LFC surface (Figures 4(a) and 4(b)). Elemental mapping analyses revealed that TiO_2_ distributed throughout the surface of the LFC substrate (Figures 4(c) and 4(d)). This is very meaningful to a floating catalyst that helps the catalyst stay active irrespective of the catalyst surface that receives the sunlight.

### 3.2. Photocatalytic Degradation toward 2,4-D

**Figure 5:**
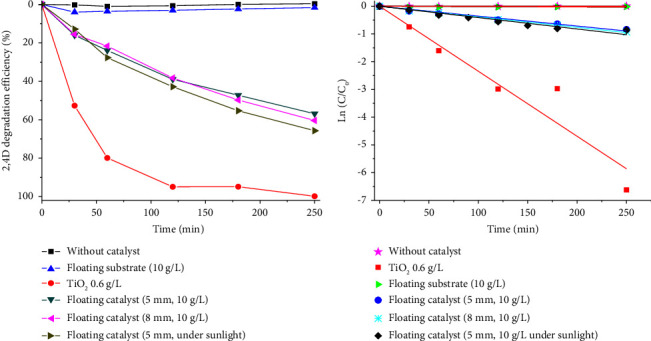
Photocatalytic behavior of photocatalysts toward 2,4-D: (a) removal efficiency and (b) photocatalytic degradation kinetics. Tests were conducted with 2,4-D 0.1 mM.

2,4-D degradation efficiency by photocatalysts is exhibited in Figure 5. A negligible decrease in 2,4-D concentration was detected after 250 min UV irradiation without catalyst. The test with LFC substrate showed a 4% reduction in the first 30 min and after that no considerable change was recorded. These suggested that the photolysis of 2,4-D occurred at a relatively slow rate and that the reduction in the presence of LFC substrate was due to its adsorption on LFC. Adsorption was also observed on FPC as the tests were conducted in the dark with a 4.5% and 9.6% reduction in 2,4-D concentration after 30 and 120 min, respectively. The adsorption of 2,4-D has very important role in the performance of FPC. This allows FPC to continuously attract pollutants from the water volume onto its surface for photocatalytic decomposition while floating on the surface without vigorous mixing.

The degradation efficiency increased sharply and reached 79.91% in the initial stage of 60 min as TiO_2_ powder was used. The degradation occurred at slower rate in the later stage and reached 99.87% after 250 min. The slow degradation in the later stage is mostly due to the low 2,4-D concentration remained in the solution. This result revealed that the synthesized TiO_2_ effectively decomposed the 2,4-D under UV radiation. The photocatalytic degradation was sustained as TiO_2_ was grafted onto the floating structure of the LFC, however, at a slower rate. As seen in Figure 5(a), the 2,4-D removal efficiency reached only 21.7% after 60 min and 60.4% in 250 min. In this study, the quantity of TiO_2_ in FPC used (0.057 g) is almost double that of TiO_2_ powder (0.03 g), thus, the slow degradation rate is likely due to the less accessibility to photocatalytic sites in the floating catalyst compared with the TiO_2_ powder. As added into water, TiO_2_ particles in powder form can disperse throughout the water phase under mixing condition, thereby, 2,4-D can approach to TiO_2_ particles instantly and then easily decomposed as TiO_2_ particles are exposed to UV light. Meanwhile, the floating catalyst appears on water surface only, it takes time for 2,4-D molecules to migrate from bulk water to the surface of catalyst. This migration induces by 2,4-D concentration gradient and is rate-limiting process. The migration rate could be enhanced by the application of external forces, i.e., stirring or air bubbling; however, it could not be occurred instantly because of the long distance. Moreover, as FPC granules float on water, only about half of their surface area exposes to the light, which further limits the activity of floating catalyst. Even though the removal efficiency achieved by FPC was lower than that achieved by TiO_2_ powder, it could be used to develop a sustainable water treatment technology. This method could considerably reduce the risk of secondary contamination and be particularly suitable for the treatment of large water resources, aquaculture, and agriculture water.

2,4-D degradation kinetics was investigated using the pseudo-first-order kinetic model, as given in the following equation:

(1) $$\begin{array}{c} r=-\frac{dC}{dt}=kC\text{,} \end{array}$$

where *r* is reaction rate, *C* is 2,4-D concentration, *t* is reaction time, and *k* is pseudo-first-order rate constant. Solving equation (1) with the boundary conditions of *t* = 0, *C*_*t*_ = *C*_0_, an integration form was obtained as the following equation:

(2) $$\begin{array}{c} \ln\;\left(\frac{C_{t}}{C_{0}}\right)=-kt\text{.} \end{array}$$

**Figure 6:**
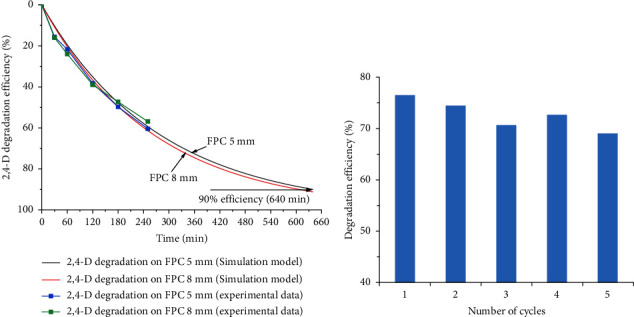
Prediction of 2,4-D degradation efficiency on FPC based on pseudo-first-order kinetic model (a) and variation in degradation efficiency after multiple cycles (b).

The rate constant, *k*, can be determined by a linear plot of Ln (*C*_*t*_/*C*_0_) vs. time (*t*), as shown in Figure 5(b). *R* square and *k* values received from linear fitting are exhibited in Table 2. Rate constants were very small, only 1.98 × 10^−6^ min^−1^ and 1.34 × 10^−4^ min^−1^, in the case no catalyst and LFC were used in the experiments, respectively. Besides, the regression is very bad for those two cases with the correlation coefficients (*R*^2^) are −0.1967 and 0.32 only. Meanwhile, the degradation rate constant for TiO_2_ powder was relatively high, reached 0.023 min^−1^ with *R*^2^ of 0.9589. Rate constants were 0.0036 min^−1^ and 0.0038 min^−1^ for FPC with granular sizes of 5 mm and 8 mm, respectively. The very close rate constants revealed that the variation in granule size from 5 to 8 mm caused no significant influence on their catalytic efficiency. The correlation coefficients reached 0.9878 and 0.9967 for FPC with granular sizes of 5 mm and 8 mm, respectively, indicated that the 2,4-D degradation on FPC fits well to the pseudo-first-order kinetic model. Simulation on 2,4-D degradation efficiency vs. time based on the pseudo-first-order kinetic model is presented in Figure 6(a). 2,4-D degradation trend resulted from the model is correlated well with that obtained from experiment. Accordingly, 90% of 2,4-D is expected to be decomposed in 640 min, equivalent to less than two sunny days depending on the location.

For large water resources such as agriculture, aquaculture, or reserve water resources, they may not require a significantly rapid treatment but rather a sustainable treatment method, and therefore, the application of the FPC could become suitable. However, to apply for this purpose, FPC must be active under the sunlight instead of UV light in the laboratory. In a previous work conducted by Shavisi et al., a floating catalyst based on P25 TiO_2_ grafted lightweight expanded clay aggregates proved to efficient candidate for NH_4_^+^ degradation under solar radiation with 96.5% NH_4_^+^ removal [39]. By grafting TiO_2_ synthesized from TTIP by the sol-gel method on palm trunk, Sboui et al. received a floating catalyst that can remove 98.2% Congo red after 210 min under solar radiation [38]. Several others demonstrated that the efficient degradation of organic compounds under sunlight can be achieved by grafting TiO_2_ on a floating substrate for floating catalyst production [33, 35–37, 41, 44, 57].

To investigate the catalytic activity of FPC prepared in this study under sunlight, experiments has been conducted in the same protocol in laboratory except the light source was changed to natural sunlight with the measured radiation power of 6.71 mW/m^2^. The result revealed that over 50% of 2,4-D was decomposed after 250 min. To evaluate the recyclability, photocatalysts were recovered, slightly washed, and dried before dispersing on water for another testing cycle. The performance of photocatalysts was assessed based on the change in degradation efficiency against 2,4-D after each cycle. Results obtained revealed that a negligible reduction in degradation efficiency (∼7.2%) was observed after 5 cycles (Figure 6(b)). This indicated that FPC is stable in experimental conditions in the laboratory. This work provided solid evidence to further confirm that FPWT could become a promising technology for water treatment.

**Figure 7:**
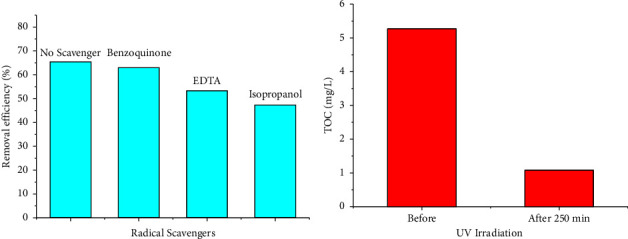
Effect of radical scavengers on the degradation of 2,4-D (a) and the variation of TOC in solution before and after 250 min under UV radiation (b).

It is well known that the radicals such as OH, O^−^_2_, h^+^ generated during UV light irradiation are responsible for the photodegradation of 2,4-D. To elucidate the role of those radicals on the photodegradation, radical scavengers—benzoquinone, EDTA, and isopropanol— were used as scavengers to capture O^−^_2_, h^+^, and OH, respectively. Experimental results showed that 2,4-D degradation efficiency slightly changes when benzoquinone was added, while the effect was significant as EDTA and isopropanol were used (Figure 7(a)). The 2,4-D degradation efficiency was reduced from 66% to 53.3% and 47.3% with the addition of EDTA and isopropanol, respectively. These results imply that h^+^ and OH radicals are the most influential radical on the 2,4-D degradation. This observation is in good agreement with a previous study where the contribution of ∙OH is dominant after 50 min irradiation on TiO_2_/activated carbon system [21]. This suggests a mechanism for 2,4-D degradation over FPC, as described in equations (3)–(9). Under UV light, TiO_2_ generates electrons and holes, which subsequently react with H_2_O and O_2_, to produce ∙OH and ∙O^−^_2_ radicals. The radicals and h^+^ can oxidize 2,4-D molecules. Total organic carbon contents in the samples decreased significantly after UV irradiation (Figure 7(b)) indicating that 2,4-D was mineralized to CO_2_ and H_2_O.

(3) $$\begin{array}{c} {TiO}_{2}+hv\;⟶\;e_{CB}^{-}+h^{+} \end{array}$$

(4) $$\begin{array}{c} h^{+}+H_{2}O\;⟶∙OH+H^{+} \end{array}$$

(5) $$\begin{array}{c} h^{+}+{OH}^{-}\;⟶∙OH \end{array}$$

(6) $$\begin{array}{c} e_{CB}^{-}+O_{2}\;⟶∙O_{2}^{-} \end{array}$$

(7) $$\begin{array}{c} ∙OH+2,4-D+O_{2}\;⟶\;de\;grada\;tion\;produ\;ct\;\left({CO}_{2}+H_{2}O\right) \end{array}$$

(8) $$\begin{array}{c} h^{+}+2,4-D\;⟶\;de\;grada\;tion\;produ\;ct\;\left({CO}_{2}+H_{2}O\right) \end{array}$$

(9) $$\begin{array}{c} ∙O_{2}+2,4-D\;⟶\;de\;grada\;tion\;produ\;ct\;\left({CO}_{2}+H_{2}O\right) \end{array}$$

## 4. Conclusion

A floating photocatalyst was successfully prepared by grafting TiO_2_ on lightweight fired clay. In this study, TiO_2_ nanoparticles were synthesized from TTIP by a hydrothermal process and grafted on floating substrate by an adsorption/calcination method. The resulting floating photocatalyst showed a great catalytic activity against 2,4-D with the degradation efficiency of 60% and 50% in 250 min under a UV radiation and sunlight, respectively. The photocatalytic degradation of 2,4-D on the floating catalyst is fitted well to the pseudo-first-order kinetic model with correlation coefficient (*R*^2^) of 0.9878 and 0.9967 and rate constant (*k*) of 0.0036 min^−1^and 0.0038 min^−1^ for catalyst granule with size 5 mm and 8 mm, respectively. Calculation based on the pseudo-first-order kinetic model indicated that 90% of 2,4-D can be treated within two days by sunlight using the floating catalyst. This demonstrated that a floating photocatalyst-based water treatment technology could be a feasible technology to degrade 2,4-D in water. The success of the work recommended a green approach to treat organic contaminants in large water resources, where they could not be treated efficiently by conventional technologies.

## Figures and Tables

**Table 1:** Chemical composition of clay.

No.	Chemical composition	Percentage (wt %)
1	SiO_2_	72.77
2	Al_2_O_3_	16.96
3	MgCO_3_	4.55
4	K_2_O	1.95
6	CaCO_3_	0.63
7	TiO_2_	0.79
8	Fe_2_O_3_	2.32
9	Others	0.13

**Table 2:** Correlation coefficients (*R*^2^) and rate constant (*k*) for photocatalytic degradation of 2,4-D over different catalysts.

Catalyst	*R* ^2^	k (min^−1^)
TiO_2_	0.9589	0.023
FPC 5 mm	0.9878	0.0036
FPC 8 mm	0.9967	0.0038
FPC 5 mm	0.9848	0.0042

## Data Availability

The data used to support the findings of this study are available within the article.
